# Anterior abdominal wall defects and their management outcomes in Tikur Anbessa Specialized Hospital, neonatal intensive care unit

**DOI:** 10.1002/jpr3.12110

**Published:** 2024-07-10

**Authors:** Abebe Habtamu, Tenagne Million

**Affiliations:** ^1^ Department of Pediatrics and Child Health, College of Health Sciences Addis Ababa University Addis Ababa Ethiopia; ^2^ Private Health Facility (Meron Clinic) Addis Ababa Ethiopia

**Keywords:** abdominal wall defect, gastroschisis, Omphalocele

## Abstract

**Introduction:**

Gastroschisis and omphalocele are common malformations of the anterior abdominal wall and can lead to significant morbidity and mortality in neonates. Despite advances in surgical care, these conditions remain challenging to manage effectively.

**Objective:**

This retrospective institutional study aimed to assess the management outcomes of neonates with anterior abdominal wall defects, specifically gastroschisis and omphalocele, in the Neonatal Intensive Care Unit (NICU) of Tikur Anbessa Specialized Hospital (TASH) (Tertiary Hospital).

**Method:**

A retrospective review was conducted on cases of gastroschisis and omphalocele managed in the NICU of TASH from August 2018 to August 2022. Patient charts of neonates with omphalocele and gastroschisis were identified and retrieved from the NICU records. The collected data were analyzed using statistical software such as SPSS.

**Results:**

The study included a total of 50 neonates with abdominal wall defects, consisting of 39 cases of omphalocele and 11 cases of gastroschisis. Maternal age ranged from 18 to 40 years, with a mean age of 27.6 ± 4.5 years. Associated malformations were documented in 33.3% of omphalocele cases and 18.2% of gastroschisis cases. Cardiac anomalies were the most frequently associated malformation with omphalocele. Surgical intervention was performed in 27.3% of gastroschisis cases (3 out of 11) and 41% of omphalocele cases (16 out of 39). The postsurgery mortality rate was 12.5% for both major and minor omphaloceles, with 11 deaths occurring in gastroschisis cases. Sepsis was identified as the cause of death in all neonates who did not survive.

**Conclusions:**

The study revealed a significantly higher mortality rate in gastroschisis cases compared to omphalocele cases. Sepsis was identified as the primary cause of death in the neonates. These findings underscore the importance of effective management strategies to prevent and manage sepsis in neonates with abdominal wall defects.

## Abstract (highlights)

**What is Known:**

- It is well known that abdominal wall defects (AAWD) are one of significant causes of morbidity and mortality even in developed countries due to the fact that most of them are associated with chromosomal anomalies.
- Feeding in neonates with AAWD is difficult mandating total parenteral nutrition.

**What is New:**

- The outcomes of this research can help to understand in utero and ante‐partal diagnosis of AAWD skills.
- Etiology or cause remains controversial, hence, can be a starting point for investigation.
- Can be useful clue to look for associated anomalies for neonatal survival.
- This study can also help to design a method for enteral and parenteral feeding methods.

## 1 INTRODUCTION

Abdominal wall defects (AAWD), specifically gastroschisis and Omphalocele, are major causes of morbidity and mortality in neonates. While survival rates have improved in developed countries due to advancements in diagnosis, surgical care, and supportive interventions,^1^ there are still controversies surrounding the etiology, anatomy, embryology, prenatal diagnosis, mode of delivery, and initial management of these conditions.^2^, ^3^, ^4^ The pathogenesis of Omphalocele is believed to be related to the formation of the anterior abdominal wall and the return of the midgut into the abdominal cavity during embryonic development.^5^, ^6^

Factors that can influence the outcomes of gastroschisis and Omphalocele include the size of the defect, prematurity, and associated congenital anomalies. Survival rates are highest when there are no associated anomalies and a normal karyotype.^7^ Mortality rates in Omphalocele can be as high as 80% when there are cardiac abnormalities, whereas cases without cardiac abnormalities have a survival rate of up to 70%.^1^

There is a significant disparity in survival rates between high‐income and low‐income countries, with higher rates observed in high‐income countries.^8^ Risk factors for adverse outcomes in low‐income countries include delivery outside of tertiary centers, lack of prenatal diagnosis, prematurity, low birth weight, sepsis, and delayed surgery.^9^ The length of time taken to achieve full enteral feedings can also impact the outcomes, with gastroschisis cases being more affected due to a slower return of intestinal motility and increased susceptibility to infections associated with indwelling lines for total parenteral nutrition.^8^

## 2 METHODS

A retrospective review of cases of Omphalocele and gastroschisis managed by the Neonatal Intensive Care Unit (NICU) within a specific timeframe. Data on patient demographics, clinical condition at presentation, interventions performed, and outcomes were collected from patient charts. Statistical analysis was conducted using SPSS software, employing mean, standard deviation, percentages, and chi‐square tests to evaluate associations between variables.

## 3 INCLUSION AND EXCLUSION CRITERIA

### 3.1 Inclusion criteria

1. All neonates admitted in NICU during the study period (August 2018–August 2022).
2. Neonates with complete neonatal chart (Biodata, surgical intervention etc.).

### 3.2 Exclusion criteria

1. Lost chart or incomplete biodata.
2. Surgical intervention incompletely documented or not documented.

### 3.3 Ethical clearance

Ethical clearance was obtained from the department research ethical committee and the Addis Ababa University Medical Faculty College of Health Sciences Institutional Review Board (IRB). A formal letter was written to the registrar office from the Department of Pediatrics and Child Health to get permission to retrieve the charts.

## 4 RESULTS

### 4.1 Study population

During the study period, a total of 14,805 children were treated by the Neonatal Intensive Care Unit (NICU) at Tikur Anbessa Specialized Hospital (TASH). Out of these cases, there were 39 cases of Omphalocele and 11 cases of gastroschisis. There were no significant differences in the number of cases of both defects over the 5‐year study period.

### 4.2 Maternal characteristics

The recorded maternal age ranged from 18 to 40 years, with a mean age of 27.6 ± 4.5 years. The parity ranged from 1 to 6 children, with a mean parity of 2.26 ± 1.34. The maternal age of gastroschisis cases was younger than that of the Omphalocele group, but the difference was not statistically significant.

### 4.3 Prenatal care and ultrasound

Among the cases, 96% of the mothers were antenatally booked, but only 6% had at least one ultrasound scan during pregnancy. Most of the patients (94%) had no documented prenatal ultrasound during pregnancy. Only 4% of the cases were suspected to have an AAWD, but the specific type of defect was not identified (Table 1).

**Table 1:** Basic characteristics of the study populations.

Characteristics	Omphalocele (*N* = 39)		Gastroschisis (*N* = 11)		*p*‐Value
	Frequency	Percent	Frequency	Percent	
Gestational age					
Term	34	87.2	8	72.7	0.344
Preterm	5	12.8	3	27.3	0.721
Place of delivery					
TAH	3	7.7	1	9.1	
Other hospitals	19	48.7	7	63.6	
Health center	6	15.4	3	27.3	
Private clinic	4	10.2	0	0	
Home	6	15.4	0	0	
Mode of delivery					0.986
SVD	32	82.1	9	81.8	
C/S	7	17.9	2	18.2	
Gender (male: female)	1:1.64		2.67:1		0.248
Birth weight (g)	2791.8 ± 616.3		2118.6 ± 600.9		0.184
Maternal age (year)	28.5 ± 4.4		24.4 ± 3.6		0.002

Abbreviations: C/S, Cesarean section; SVD, spontaneous vertex delivery; TAH, Tikur Anbessa Hospital.

### 4.4 Place of delivery and mode of delivery

The majority of patients (34%) were delivered in governmental hospitals other than TASH, followed by private hospitals (18%). Home delivery accounted for 12% of cases, and health centers accounted for 18%. Most neonates were delivered by normal spontaneous vertex delivery, with 64% of Omphalocele cases and 18% of gastroschisis cases delivered in this manner. Cesarean section was performed in 18% of Omphalocele cases and 4% of gastroschisis cases. There was no statistically significant difference in the mode of delivery between the two groups (Table 1).

### 4.5 Associated congenital anomalies

Thirteen out of 39 neonates with Omphalocele (33.3%) had associated congenital anomalies, while only 2 out of 11 neonates with gastroschisis had associated anomalies. Cardiac anomalies were the most frequently associated anomalies in Omphalocele cases (54%). Two cases of gastroschisis had intestinal atresia. The type of associated anomaly had a statistically significant association with the type of AAWD (Table 2).

**Table 2:** Shows associated congenital anomalies.

Type of congenital anomaly detected	Type of AAWD			
	Omphalocele		Gastroschisis	
	Frequency	Percent	Frequency	Percent
Cardiac	7	18	0	0
Genitourinary	4	10.0	0	0
Abdomen	0	0	2	18.2
Limb	2	5.1	0	0
Suspected syndromes and chromosomal anomalies	3	7.7	0	0
No anomaly	23	59	9	81.8
Total	39	100	11	100

Abbreviation: AAWD, abdominal wall defects.

### 4.6 Surgical management and complications

Surgery was performed in three cases (27.2%) of gastroschisis and 16 cases (41%) of Omphalocele. Primary closure without prosthesis was done in all 16 Omphalocele cases, with 14 cases undergoing fascial closure and one case undergoing skin closure. Primary fascial closure was done for two gastroschisis cases, and primary skin closure was done for one case. The postoperative complication rate was 10.5%, with all complications attributed to sepsis and wound infection. The postsurgery mortality rate was 12.5% for both major and minor Omphaloceles, and one patient with gastroschisis died after surgery.

### 4.7 Nonoperative management and mortality

Nonoperative management of the defect by the use of antiseptic and sac dressing was done in 31 patients, with 23 cases of Omphalocele and eight cases of gastroschisis managed conservatively. The mortality rate was 18% for all Omphaloceles, 30.4% for major Omphaloceles, and 26.1% for unruptured Omphaloceles. Patients with gastroschisis managed conservatively had a mortality rate of 72.7%.

The length of hospital stay ranged from 1 to 31 days, with a mean of 9 ± 7.58 days.

The presence of sepsis, hypothermia, wound infection, and jaundice at presentation, as well as the mode of management (conservative or surgical), had a statistically significant association with the outcome. The overall mortality rate was 38%, and the documented cause of death for all cases was sepsis.

## 5 DISCUSSION

### 5.1 Prevalence and lack of data

The estimated birth prevalence of Omphalocele and gastroschisis in western countries is relatively low but varies in different regions.^6^, ^10^, ^11^, ^12^, ^13^, ^14^, ^15^ The true prevalence of these conditions in Africa, particularly in sub‐Saharan Africa including Ethiopia, is not known due to the lack of population‐based studies, central birth registers, and prenatal diagnosis.^2^, ^5^, ^16^ This suggests a need for more comprehensive data collection and research to better understand the prevalence and impact of these conditions in African countries.

### 5.2 Maternal age and antenatal care

The maternal age of gastroschisis cases in the study tended to be younger, although not statistically significant.

The majority of mothers had antenatal care (ANC) follow‐up, but only a small percentage had documented prenatal ultrasounds, which are crucial for detecting these conditions. Lack of access to scanning equipment, trained sonographers, and fetal medicine specialists were identified as potential reasons for the low antenatal diagnosis rates.^17^, ^18^, ^19^ The study emphasizes the importance of increasing awareness and access to prenatal ultrasound for early detection and appropriate management.

### 5.3 Associated anomalies

The study found that a significant proportion of cases had associated anomalies, with Omphalocele cases having a higher rate than gastroschisis cases. Cardiac anomalies were common in Omphalocele cases, while intestinal atresia was observed in some gastroschisis cases. The incidence of associated anomalies varied across different studies, suggesting the need for comprehensive evaluations and screenings for associated conditions in affected infants.

### 5.4 Mortality rates

The overall mortality rate in the study was high, with the highest case fatality observed in gastroschisis cases. The documented cause of death for all deceased patients was sepsis, indicating the importance of infection prevention and early management. The mortality rates reported in the study were higher compared to some previous studies, emphasizing the need for improved care and interventions to reduce mortality in these cases.

## 6 RECOMMENDATIONS

The study highlights the need to increase awareness and improve the diagnosis of AAWDs through prenatal ultrasound. Early diagnosis is crucial for appropriate management and delivery at specialized medical institutions.

The high mortality rates, primarily due to sepsis, emphasize the importance of early detection, infection prevention, and timely interventions to improve outcomes.

## 7 CONCLUSION

The study underscores the challenges faced in diagnosing and managing Omphalocele and gastroschisis in Ethiopia. It emphasizes the need for improved prenatal care, access to ultrasound facilities, and the development of strategies to reduce mortality rates associated with these conditions.

## CONFLICT OF INTEREST STATEMENT

The authors declare no conflict of interest.
